# Differences in intracellular localisation of ANKH mutants that relate to mechanisms of calcium pyrophosphate deposition disease and craniometaphyseal dysplasia

**DOI:** 10.1038/s41598-020-63911-x

**Published:** 2020-05-04

**Authors:** Sunny Vijen, Chris Hawes, John Runions, R. Graham G. Russell, B. Paul Wordsworth, Andrew J. Carr, Ryan C. Pink, Yun Zhang

**Affiliations:** 1 Department of Biology and Medical Sciences, Oxford Brookes University, Gipsy Lane, Oxford, OX3 0BP UK; 2 Nuffield Department of Orthopaedics, Rheumatology and Musculoskeletal Sciences, University of Oxford Institute of Musculoskeletal Sciences, Windmill Road, Oxford, OX3 7HE UK

**Keywords:** Disease genetics, Rheumatology, Autophagy, Mechanisms of disease

## Abstract

ANKH mutations are associated with calcium pyrophosphate deposition disease and craniometaphyseal dysplasia. This study investigated the effects of these ANKH mutants on cellular localisation and associated biochemistry. We generated four ANKH overexpression-plasmids containing either calcium pyrophosphate deposition disease or craniometaphyseal dysplasia linked mutations: P5L, E490del and S375del, G389R. They were transfected into CH-8 articular chondrocytes and HEK293 cells. The ANKH mutants dynamic differential localisations were imaged and we investigated the interactions with the autophagy marker LC3. Extracellular inorganic pyrophosphate, mineralization, ENPP1 activity expression of ENPP1, TNAP and PIT-1 were measured. P5L delayed cell membrane localisation but once recruited into the membrane it increased extracellular inorganic pyrophosphate, mineralization, and ENPP1 activity. E490del remained mostly cytoplasmic, forming punctate co-localisations with LC3, increased mineralization, ENPP1 and ENPP1 activity with an initial but unsustained increase in TNAP and PIT-1. S375del trended to decrease extracellular inorganic pyrophosphate, increase mineralization. G389R delayed cell membrane localisation, trended to decrease extracellular inorganic pyrophosphate, increased mineralization and co-localised with LC3. Our results demonstrate a link between pathological localisation of ANKH mutants with different degrees in mineralization. Furthermore, mutant ANKH functions are related to synthesis of defective proteins, inorganic pyrophosphate transport, ENPP1 activity and expression of ENPP1, TNAP and PIT-1.

## Introduction

Under normal conditions cartilage and bone calcification involves  hydroxyapatite (HA) deposition requiring the non-enzymatic reaction of extracellular calcium and inorganic phosphate (ePi). In rheumatologic conditions like calcium pyrophosphate dihydrate deposition disease (CPPDD) calcium pyrophosphate dihydrate (CPPD) can deposit contributed by Inorganic pyrophosphate (PPi), a regulator of calcification, inhibiting HA deposition. At least four proteins influence PPi homeostasis in the extracellular matrix (ECM): these include the transmembrane pyrophosphate transporter ANKH (the human homologue to the mouse progressive ankylosis protein), ENPP1 (ectonucleotide pyrophosphatase 1), TNAP (tissue nonspecific alkaline phosphatase, one of the four forms of alkaline phosphatase ALPL) and PIT-1 (sodium-dependent phosphate transport 1, also named as SLC20A1); ENPP1 readily hydrolyses extracellular adenosine triphosphate (eATP) into extracellular PPi (ePPi) and extracellular AMP (eAMP), whilst ANKH exports intracellular PPi (iPPi) into the ECM, TNAP hydrolyses ePPi into extracellular Pi(ePi), and PIT-1 imports ePi into the cell via a sodium exchange channel to be used in metabolic pathways^1,2^.

ANKH is mainly expressed at the cell membrane and mutations in *ANKH* cause two distinct conditions - CPPDD [MIM118600] and craniometaphyseal dysplasia (CMD [MIM123000])^3,4^. CPPDD typically presents with destructive arthritis and may mimic rheumatoid arthritis, gout or osteoarthritis and is the commonest form of inflammatory monoarthritis in the elderly, occurring in up to 40% of those over 65 years of age^1,5^. In contrast, CMD is a rare disorder characterised by hyperostosis/sclerosis of the skull and abnormal modelling of the long bones, and individuals with severe forms of CMD can have reduced life expectancy as a result of compression of the foramen magnum^4^. CMD is associated with decreased ePPi, which allows increased HA deposition and altered bone modelling via chondrogenesis, osteoblastogenesis and osteoclastogenesis^6^. There is currently no specific treatment for CPPDD and CMD.

Mutations near either end of *ANKH* are mostly associated with CPPDD while mutations in the middle have been reported to cause CMD, though their biological effect and cellular function remain largely unexplored. Previous research shows CPPDD associated P5L (p.Pro5Leu, NM_054027.4:c.14 C > T) to increase expression of ANKH and was reported to increase expression and activity of ENPP1^7,8^. E490del (p.Glu490del, NM_054027.4:c.1468_1470delGAG) deregulated TNAP activity^9,10^. CMD related mutants have largely been restricted to clinical case studies. One case report of S375del (p.Ser375del, NM_054027.4:c.1123_1125delTCC) showed a decrease in ePPi that was consistent with the predicted loss-of-function^11^. G389R (p.Gly389Arg, NM_054027.4:c.1165 G > A), reported in several cases of CMD and recently in CPPDD, where it was predicted to be a loss-of-function variant^4,12^.

Autophagy is a dynamic catabolic mechanism that recycles damaged organelles and non-functional proteins and maintains cellular homeostasis^13^. Previous studies have highlighted the importance of autophagy and its modulation of genes in maintaining healthy chondrocytes in the formation of cartilage and preventing degeneration during osteoarthritis^14,15^.

To investigate the pathogenic mechanisms of ANKH in CPPDD and CMD, we generated four disease-associated ANKH mutants associated with relatively severe clinical phenotypes: two are terminally positioned P5L and E490del associated with CPPDD, two are centrally positioned S375del and G389R associated with CMD. We used confocal imaging to identify ANKH mutant cell localisation dynamics, measured ePPi concentrations and altered mineralization level, evaluated ANKH mutant effect on the function of ENPP1 and gene expression of *ENPP1*, *TNAP* and *PIT-1.* We also investigated the involvement of autophagy for potential mutated ANKH protein recycling in the pathogenesis of CPPDD and CMD.

## Results

We found that ANKH mutations altered cellular localisation dynamics and led to biochemical changes at different levels by comparing with wt.ANKH. Our detailed findings are summarised in Table 1 and the details are described as below.

**Table 1:** Summary of ANKH mutant effects.

	Time (H)	HEK293					CH-8	
		Cells with membrane expression (n > 100)	ePPi (µM)	*Expression* (fold change)			Mineralization (nM)	ENPP1 Activity (fold change)
				*ENPP1*	*TNAP*	*PIT-1*		
wt.ANKH^a^	24	57%	NA	0.49^#^	0.56	0.79		0.7**
	48	66%	2.6*	2.4*	1.28	1.29		1.93**
	72	66%	3.2*	1.6*	0.61	2.32*		1.05
	96						37.15**	
P5L^b^	24	34%	NA	1.48	1.72*	1.02		1.92**
	48	64%	2.2	1.38	0.76	1.26		1.54**
	72	73%	6.9*	1.11	1.39^#^	1.12		3.39**
	96						44.64**	
S375del^b^	24	0%	NA	1.62^#^	1.95^#^	0.93		1.14**
	48	0%	3.1	0.91	0.72	1.88*		0.65**
	72	0%	1.8^#^	1.06	1.21	0.89		1.02
	96						52.68**	
G389R^b^	24	28%	NA	2.28*	1.08	1.22		1.93**
	48	66%	1.3	0.64	0.65	1.39		0.59**
	72	72%	2.6	0.72	1.26	0.74		0.74**
	96						40.62**	
E490del^b^	24	35%	NA	5.82**	2.79**	2.13*		5.33**
	48	7%	1.1	0.89	0.86	1.77^#^		0.85
	72	29%	3.7	0.8	1.07	0.85		1.01
	96						42.79**	

^a^Fold change of *wt. ANKH* is compared to null vector controls, ^b^mutant *ANKH* to *wt*. *ANKH*. H: hours after transfection/induction of mineralization. *p value < 0.05, **p value < 0.01, ^#^*p value* 0.05 < 0.1.

### Wt.ANKH localisation to the cell membrane and its influence on the expression levels of ENPPI, TNAP and PIT-1

**Figure 1:**
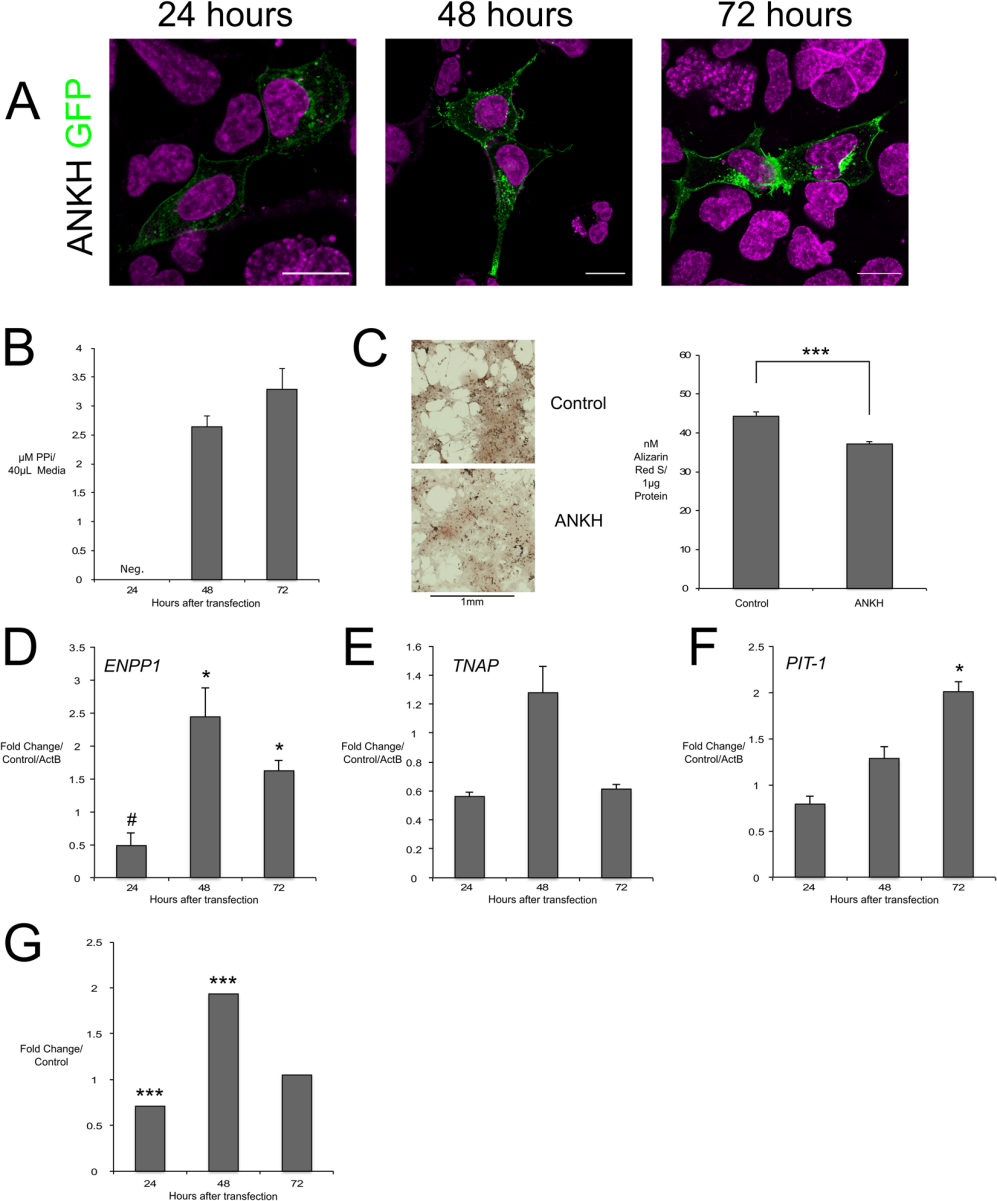
Wt.ANKH overexpression intracellular localisation and biochemical effects of overexpression compared to null vector control in CH-8 and HEK293 cells. Wt.ANKH-GFP (green) in HEK293 cell membrane (**A**); increased ePPi in HEK293 culture media (**B**); Alizarin red S staining of CH-8 cells (left) and concentration of ARS/µg protein (right) normalized to ActB confirming reduction in mineralization (**C**); qPCR in transfected HEK293 cells trended to decrease ENPP1 at 24 H and increase at 48 and 72 H (**D**), did not significantly change TNAP (**E**), increased PIT-1 72 H (**F**); ENPP1 activity in CH-8 cells decreased at 24 H and increased at 48 H (**G**). H = hours, scale bars = 20 μm; *** indicates *p* value < 0.01; * indicates p value < 0.05. # indicates *p* value < 0.1; neg. = Negligible/Raw value below threshold.

Wt.ANKH with GFP at either the N or C terminal showed clear localisation to the cell membrane and perinuclear area in HEK293 cells as reported in other cell types such as osteoblastic MC3T3-E1, human adult fibroblasts (HAF), adenocarcinomic human alveolar basal epithelial cells (A549), HeLa and monkey Cos7 cells (Figs. 1A, S1)^16,17^. We observed this specific cell membrane localisation in the majority of transfected cells at all-time points after transfection, unlike any other mutant. Further clarification using 3D confocal Z-stack rotating rendering also showed the localisation was primarily found in the cell membrane (Movie S1). CH-8 cells displayed auto-fluorescence that masked overexpression of control eGFP and wt.ANKH-eGFP (Fig. S2 and Movie S2). Overexpressing wt.ANKH in HEK293 cells increased ePPi following 48 hours after transfection (Fig. 1B). Interestingly, wt.ANKH overexpression in CH-8 cells significantly decreased mineralization compared to GFP only control (Fig. 1C). Wt.ANKH in HEK293 cells showed gene expression of *ENPP1* significantly increased at 48 and 72 hours, peaking at 48 hours (Fig. 1D), this pattern was mirrored in the ENPP1 activity of CH-8 cells (Fig. 1G). A similar, but non-significant trend, was measured for *TNAP* at 24, and 72 hours after transfection (Fig. 1E). *PIT-1* increased significantly at 72 hours (Fig. 1F). Wt. ANKH-eGFP did not co-localise with pmRFP-LC3 under autophagic or rescue conditions (Fig. S3).

### P5L ANKH slowed initial membrane recruitment and increased ePPi and mineralization via increased ENPP1 activity

**Figure 2:**
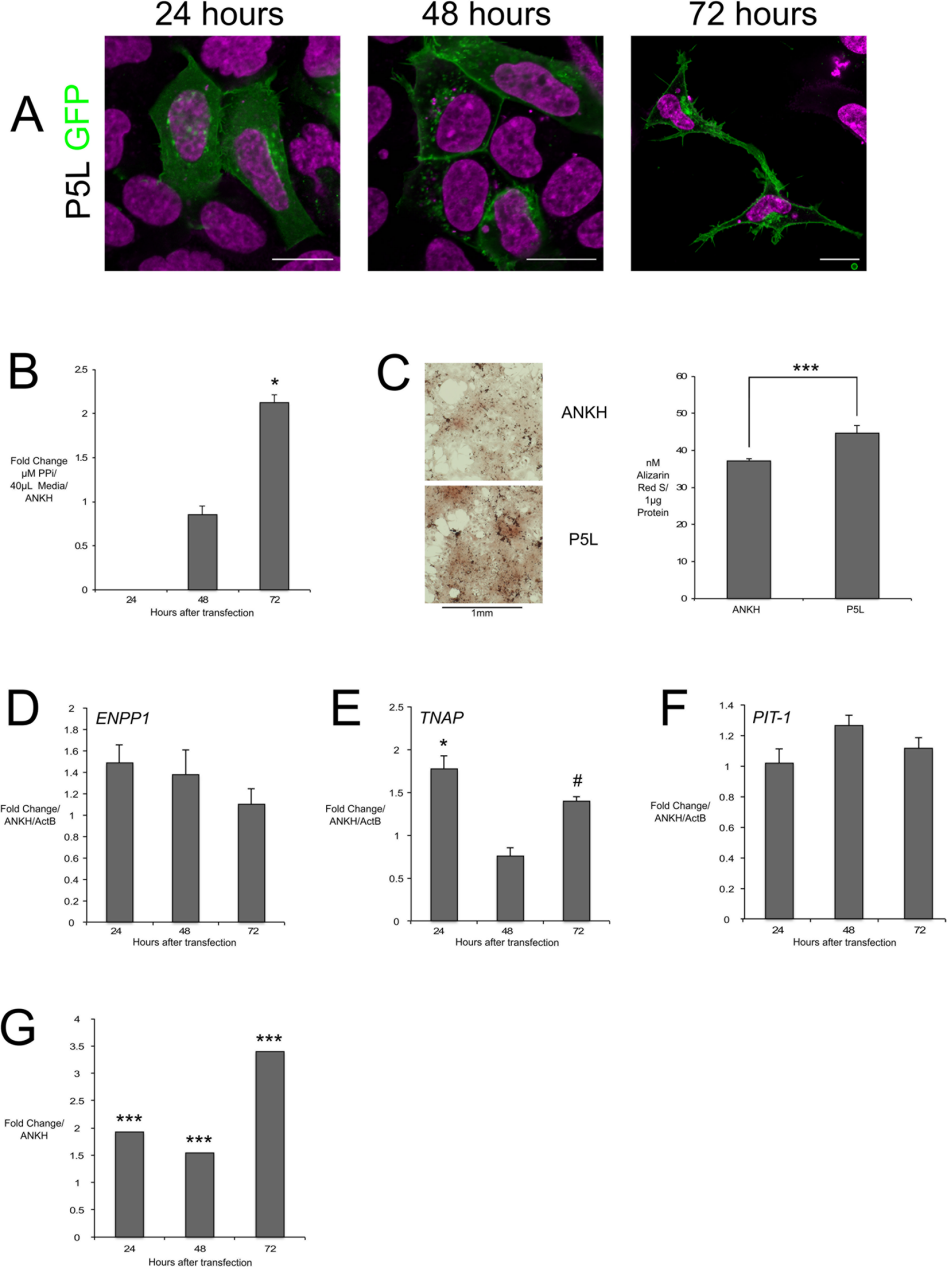
P5L overexpression intracellular localisation and biochemical effects compared to wt.ANKH control in CH-8 and HEK293 cells. P5L-GFP (green) mainly localised to HEK293 cytoplasm at 24 H and cell membrane at 72 H (**A**); increased ePPi in HEK293 culture media (**B**); ARS staining of CH-8 cells (left) and concentration of ARS/µg protein (right) normalized to ActB confirming increased mineralization (**C**); no significant change in ENPP1 (**D**), significant increased TNAP at 24 H and trended increase at 72 H (**E**), no significant change in PIT-1 (**F**); ENPP1 activity in CH-8 cells increased at 24, 48 and 72 H (**G**). H = hours, scale bars = 20 μm; *** indicates *p* value < 0.01; * indicates p value < 0.05. # indicates *p* value < 0.1; neg. = Negligible/Raw value below threshold.

We observed an initial diffuse expression of P5L within the cytoplasm with perinuclear aggregation and formation of vesicle like structures at 24 hours, with only 34% of HEK293 cells displaying specific cell membrane localisation, but returned to wild-type levels at 48 and 72 hours (Fig. 2A). P5L significantly increased ePPi double that of wild-type control at 72 hours in HEK293 cells and mineralization increased 20% in CH-8 cells (Fig. 2B,C). It also increased *TNAP* in HEK293 cells but did not change *PIT-1* (Fig. 2E,F). In CH-8 cells we observed insignificant increases in *ENPP1* expression, but ENPP1 activity as consistently high at all-time points (Fig. 2D,G).

### S375del completely abolished ANKH localisation to the cell membrane, decreased ePPi and increased mineralization

**Figure 3:**
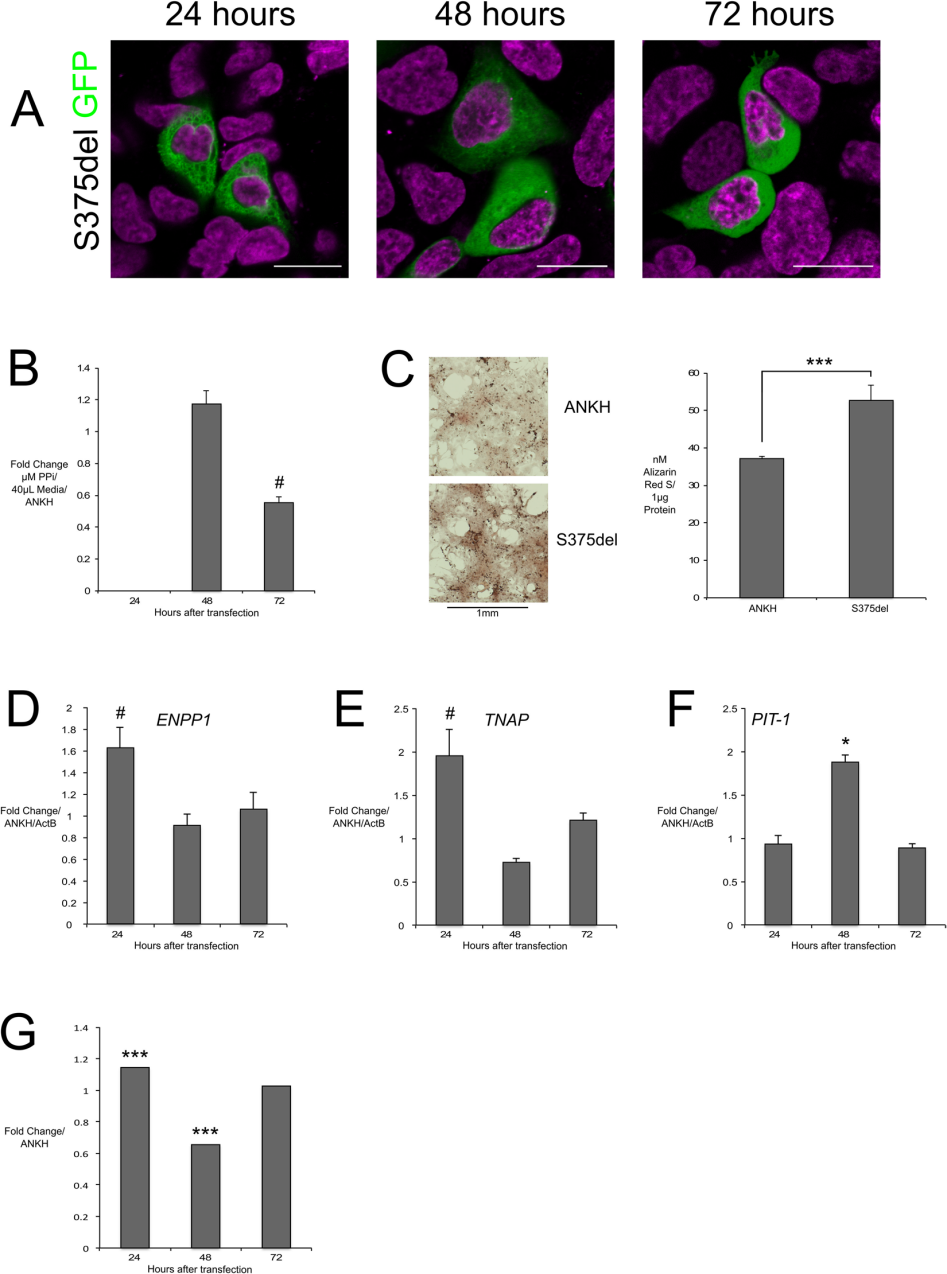
S375del overexpression intracellular localisation and biochemical effects compared to wt.ANKH control in CH-8 and HEK293 cells. S375del-GFP (green) localised to HEK293 cytoplasm at all time points (**A**); little change in ePPi concentration in culture media of HEK293 (**B**); ARS staining of CH-8 cells (left) and concentration of ARS/µg protein (right) normalized to ActB confirming increased mineralization (**C**); S375del in HEK293 cells trended to increase ENPP1 and TNAP at 24 H (**D**,**E**), significantly increased PIT-1 at 48 H (**F**); ENPP1 activity in CH-8 cells showed slightly increased at 24 H and decreased at 48 H (**G**). H = hours, scale bars = 20 μm; *** indicates *p* value < 0.01; * indicates p value < 0.05. # indicates *p* value < 0.1; neg. = Negligible/Raw value below threshold.

S375del completely rendered ANKH unable to enter the HEK293 cell membrane and remained diffusely expressed within the cytoplasm at 24, 48 and 72 hours after transfection Fig. 3A). We measured a trending decrease in ePPi at 72 hours in HEK293 cells and observed a significant 40% increase in mineralization in CH-8 cells compared to wt.ANKH (Fig. 3B,C). In HEK293 cells we measured non-significant increases in *ENPP1* and ENPP1 activity in CH-8 cells was found to increase at 24 hours but decrease at 48 hours (Fig. 3D,G). *PIT-1* increased at 48 hours (Fig. 3F).

### G389R slowed initial ANKH to insert in the membrane and trended to decrease ePPi, increased mineralization and was potentially targeted as a non-functional protein

**Figure 4:**
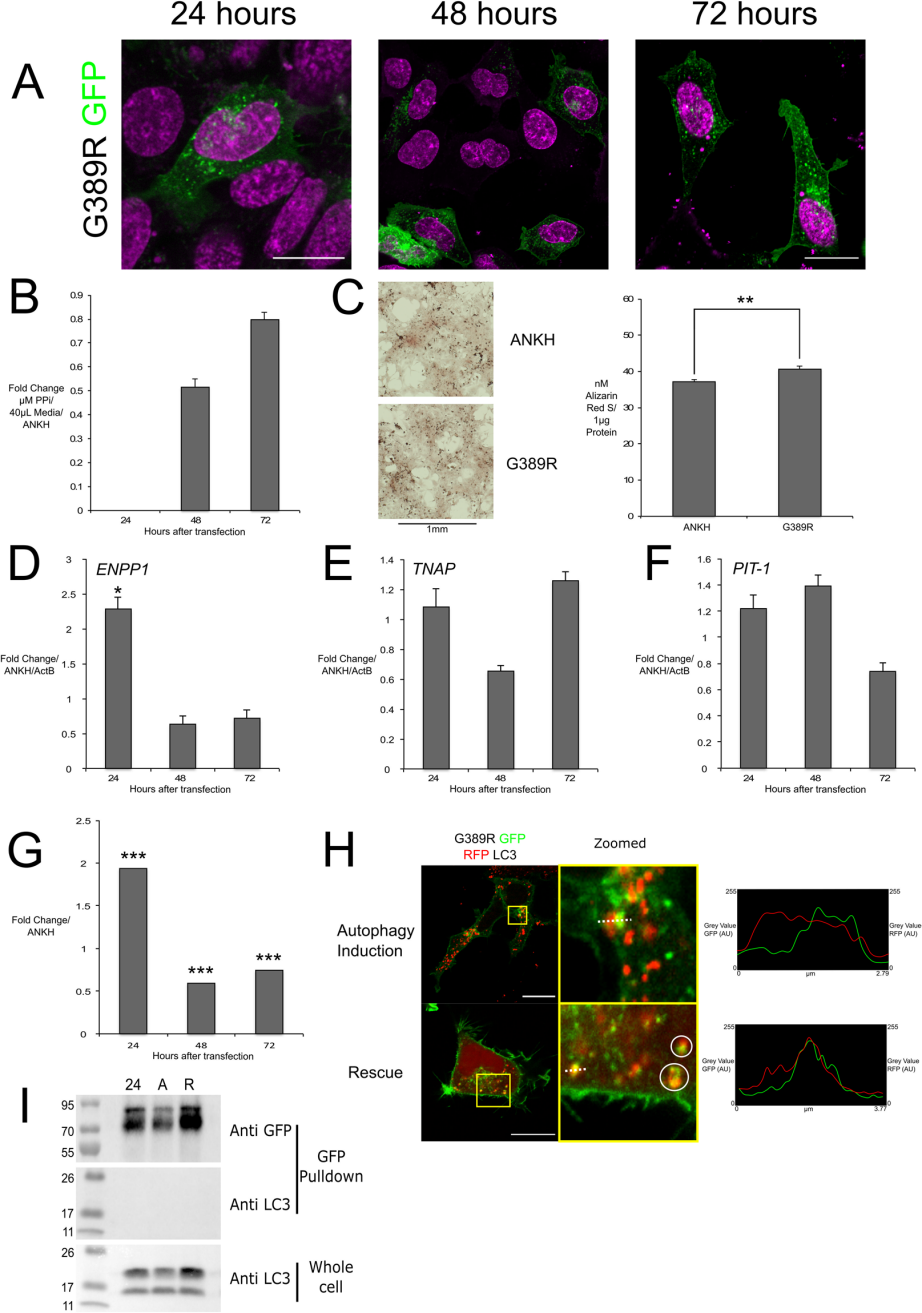
G389R overexpression intracellular localisation and biochemical effects compared to wt.ANKH control in CH-8 and HEK293 cells with no direct LC3 interaction. G389R-GFP (green) mainly localised to HEK293 cell membrane with many puncta (**A**); increased ePPi in HEK293 culture media (**B**); ARS staining of CH-8 cells (left) and concentration of ARS/µg protein (right) normalized to ActB confirming increased mineralization (**C**); G389R in HEK293 cells increased ENPP1 at 24 H (**D**), did not significantly change of TNAP and PIT-1 at any time (**E,F**), ENPP1 activity in CH-8 cells increased at 24 H, then decreased at 48–72 H (**G**); Live confocal G389R ANKH-GFP (green) and RFP-LC3 (red) showing co-localisation during autophagy induction and rescue (circled) confirmed with intensity over distance (dotted line) (**H**); HEK293 Lysates of G389R ANKH-GFP and RFP-LC3 (rep of three) at 24 H, with autophagic media for 3 h (**A**), with normal growth media for 24 H (**R**); top: anti-GFP Western blot of IP GFP pull down, shows G389R not physically interact with LC3 (**I**). H = hours, scale bars = 20 μm; *** indicates *p* value < 0.01; * indicates p value < 0.05. # indicates *p* value < 0.1; neg. = Negligible/Raw value below threshold.

G389R expressed mostly within the cytoplasm with just 28% of HEK293 cells displaying specific cell membrane localisation at 24 hours, half that of the wild-type control, but show similar localisation after 48 hours, though there was novel vesicle like puncta (Fig. 4A). In HEK293 cells we found a non-significant decrease in ePPi at 48 and 72 hours. In CH-8 cells we measured small, though significant, increases in mineralization (Fig. 4B,C). G389R overexpressed in HEK293 cells was found to significantly increase of over two-fold *ENPP1* early compared to wildtype at 24 hours (Fig. 4D), increased ENPP1 activity in CH-8 cells at 24 hours was also seen dropping from 48 hours (Fig. 4G). We also observed co-localisation of G389R-eGFP with RFP-LC3 under autophagic and rescue conditions in HEK293 cells (Fig. 4H, zoomed and intensity over distance plots and Movie S3). We did not observe co-IP of LC3 with mutant G389R (Fig. 4I).

### E490del mutated ANKH remained mostly cytoplasmic and formed punctate like structures that co-localised with LC3, and increased mineralization through increased expression of ENPP1, TNAP and PIT-1

**Figure 5:**
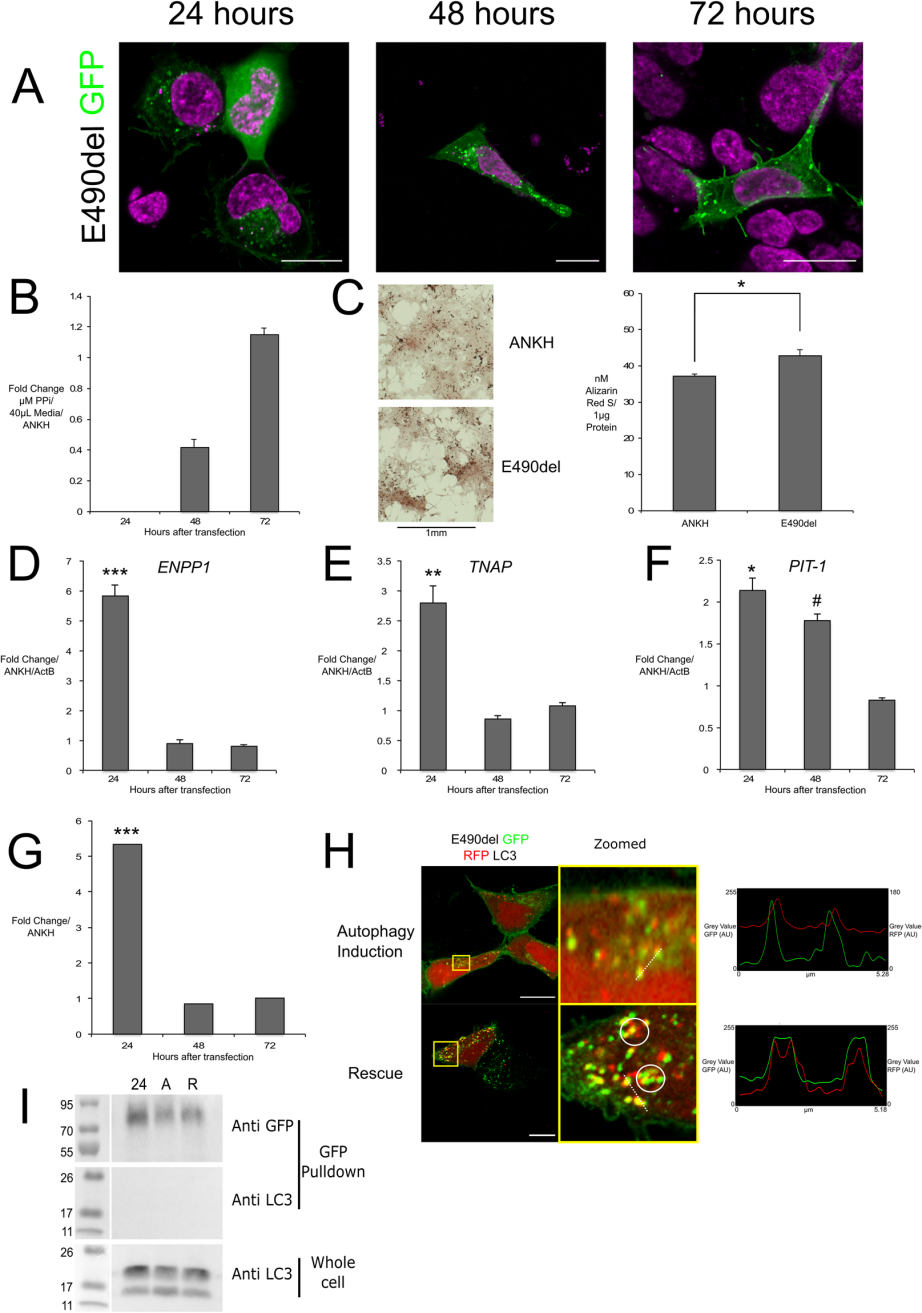
E490del overexpression intracellular localisation and biochemical effects of compared to wt.ANKH control in CH-8 and HEK293 cells with no direct LC3 interaction. E490del ANKH-GFP (green) localised mainly to cytoplasm and formed puncta at 24, at 72 H majority in membrane (**A**); increased ePPi in HEK293 culture media (**B**); ARS staining of CH-8 cells (left) and concentration of ARS/µg protein (right) normalized to ActB confirming increased mineralization (**C**); E490del in HEK293 cells increased ENPP1 and TNAP at 24 H (**D**,**E**); increased PIT-1 at 24–48 H (**F**); increased ENPP1 activity in CH-8 cells at 24 h (**G**); Live confocal E490del ANKH-GFP (green) and RFP-LC3 (red) showing co-localisation during autophagy induction and rescue (circled) confirmed with intensity over distance (dotted line) (**H**); HEK293 Lysates of E490del ANKH-GFP and RFP-LC3 (rep of three) at 24 H, with autophagic media for 3 h (**A**), with normal growth media for 24 H (**R**); top: anti-GFP Western blot of IP GFP pull down, shows E490del not physically interacting with LC3 (I). H = hours, scale bars = 20 μm; *** indicates *p* value < 0.01; * indicates p value < 0.05. # indicates *p* value < 0.1; neg. = Negligible/Raw value below threshold.

E490del localised primarily to the cytoplasm at all time points, showing less than half of the membrane expression after 72 hours of wild-type control, and like G389R formed vesicle like puncta after 48 hours (Fig. 5A). The ePPi slowly increased over time at similar levels to wild-type with no significant difference. In CH-8 cells we observed a significant 15% increase in mineralization (Fig. 5B,C). Strikingly E490del increased expression of *ENPP1* by over five-fold compared to wt.ANKH in the HEK293 cells, reflected by a five-fold increase in ENPP1 activity in CH-8 cells (Fig. 5D,G). This early expression at 24 hours is also seen in the two-fold increases of *TNAP* and *PIT-1* (Fig. 5E,F). Similar to G389R we observed co-localisation of E490del-GFP with RFP-LC3 under autophagic and rescue conditions (Fig. 5H, zoomed and intensity over distance plots and Movie S4). We did not observe co-IP of LC3 with E490del (Fig. 5I).

## Discussion

ANKH function at the cell membrane to transport PPi into the ECM is well established, however. We initially show ANKH localisation to the cell membrane to be non-homogenous and a striking distinction between the localisation dynamics of each ANKH mutants. P5L and G389R both localised more slowly to the cell membrane, E490del showed some cell membrane localisation but was mostly cytoplasmic and S375del did not at any time. A recent paper on novel mutant ANKH-F377del, also shows evidence of the mutated protein to be dispersed in the cytoplasm^18^. Although we see similar wt.ANKH localisation as reported by Kanaujiya, J. *et al*.^18^ and many other non-bone cell lines, we cannot discount the effects that this may be specific to bone. These very different localisation dynamics, even between mutants causing similar clinical features, highlights the complex and diverse function of ANKH in the pathogenesis of CPPDD and CMD.

We quantified each ANKH mutant effect on ePPi concentration and potentially non-physiological, mineralization, and as expected overexpression of wt. ANKH increased ePPi level compared to endogenous ANKH, but the CPPDD linked gain-of-function P5L caused a remarkable increase of over double ePPi compared to wt.ANKH once a majority of the mutant had inserted into the cell membrane at 72 hours, suggesting a slowed response to ANKH changes in the pathophysiology, or even limited with sustained mutation expression. Conversely, both CMD linked loss-of-function S375del and G389R trended to decrease ePPi. In the ECM ePPi plays a key role in inhibiting HA accretion and bone mineralization^2,7^. In CMD, there is thought to be abnormal skeletal modelling due to excessive HA mineralisation because of the decrease in ePPi^19^, a phenotype confirmed by our novel molecular data. In contrast, increased ePPi leads to excessive CPPD mineralization in the ECM of those suffering with CPPDD. Our data highlighted that all tested mutants had pathologically increased mineralization. Furthermore, the quantity of mineralization ranks the mutations in their severity of clinical phenotypes with S375del being the most severe, second P5L, third E490del and fourth G389R. Taken together, our data suggest a direct and novel link between differential cellular localisation, altered ePPi concentration and mineralization associated with mutated forms of ANKH.

As bone mineralization is regulated by the ratio of [Pi]/[PPi] in the growth plate ECM, a synergistic relationship between ANKH, ENPP1, TNAP and PIT-1 exists to control physiological *vs*. pathophysiological mineralization^2,20–23^. We explored some of the reported interactions of ANKH with ENPP1, TNAP or PIT-1 in regulating ePPi and discerned the complexity of these relationships, especially in the context of disease progression and causality. Increased *ENPP1* and *PIT-1* of wt.ANKH corresponded to the increase in ePPi, and the reduction in *TNAP* was concordant with decreased mineralization. Therefore, simply increasing the quantity of ANKH did not replicate a gain-of-function phenotype. This is likely due to saturation of the ANKH - ENPP1 interaction, where ENPP1 is thought to provide most of the ePPi present in the ECM^24–26^. The decrease in mineralization can be attributed to the mild increase in ePPi, enough to further inhibit HA accretion but insufficient for CPPD formation.

P5L increased expression of *TNAP* at 24 hours as a response to increased ePPi, but not sustained; however the level of *ENPP1* and *PIT-1* were similar to wt.ANKH. Interestingly, we measured an increase in ENPP1 activity at 24, 48 and 72 hours, and therefore we suggest that P5L raised ePPi via increased ENPP1 activity rather than increased ANKH function or increased *ENPP1* expression – enlightening the previous observation showing P5L to increase activity of ENPP1^8^. Furthermore, the increased ePPi and non-changed *PIT-1* indicated that the increased mineralization caused by P5L was specific to CPPD formation rather than HA. Inability of S375del to insert into the membrane was so severe that even increased expression of *ENPP1*, *TNAP*, *PIT-1* and corresponding ENPP1 activity as compensation was not enough to rescue the phenotype. S375del showed by far the largest mineralization profile and decrease in ePPi over time indicated the mineralization to be likely HA rather than CPPD. In the long term, reduced ANKH membrane presence would exacerbate a condition reliant on extracellular ePPi control and this explains the severe phenotype. The milder phenotype G389R increased expression and corresponding activity of ENPP1 but did not change *TNAP* and *PIT-1*. Interestingly, as G389R was recruited to the cell membrane similar to wt.ANKH but still increased ENPP1 activity and expression as compensation for decreasing ePPi levels, this suggested a severe reduction in transport capability for G389R but needs further exploration. E490del drastically increased early expression of *ENPP1*, *TNAP* and *PIT-1* and corresponding ENPP1 activity as compensation for lack of ANKH at the cell membrane, however as this mutant did not completely abolish ANKH localisation and hence, the compensatory effect seemed to be overpowered and might indeed be causal in the pathogenesis of CPPDD in sufferers affected by the E490del mutation, requiring further study.

Abnormal or defective proteins are often targeted by autophagic machinery for recycling^27^. Co-localisation of the autophagy marker LC3 with E490del under normal, autophagy induction and rescue conditions, and with G389R under autophagy induction and rescue conditions could suggest that these mutated proteins were targeted as non-functional or defective proteins. We also checked for direct physical interaction of ANKH with LC3, which may indicate a role in the autophagy process, however we did not observe co-IP of these mutants with LC3, making it more likely that G389R and E490del were targets of autophagy as non-functional or defective proteins rather than facilitators in the autophagy process. The absence of co-localisation for P5L and S375del does not suggest they are not recycled as this process is not limited to autophagy, for example an endoplasmic-reticulum-associated protein degradation (ERAD) ubiquitination pathway that is preferential for insoluble aggregates within the cytoplasm could explain no co-localising of S375del with LC3^27^.

We summarize the possible mechanisms in the pathogenesis of each tested mutants associated with CPPDD or CMD as follows:

### P5L

This proline to leucine mutation may delay ANKH membrane uptake and the longer term increasing in ePPi could be attributed to an overreaction from increased ENPP1 activity rather than *ENPP1* expression directly. This is likely that the rise in ePPi is not due to increased ENPP1 amounts at the cell membrane but rather to increased activity of ENPP1 that is already at the cell membrane facilitated by the P5L mutation as suggested by previous work and now also our ENPP1 activity assay. Since TNAP expression increased initially and has a role in reducing excess ePPi, we suggest that there should be a feedback mechanism to upregulate TNAP under conditions of high ePPi but cannot compensate for the mutation effect.

### S375del

Our data suggests that deletion of serine at residue 375 completely abolishes ANKH localisation to the cell membrane and is a loss-of-function. We observed almost cytoskeletal-like localisation over 72 hours and measured a corresponding decrease in ePPi and increase in mineralization. The phenotype of S375del CMD is severe and at the molecular level even the compensatory increase in *ENPP1*, *TNAP* and *PIT-1* could not rescue the biochemical phenotype. Mechanistically, we predict the serine deletion results in inappropriate trafficking from the ER and abolishes ANKH function.

### G389R

This mutant displayed equivocal molecular data highlighting its variable clinical associations with both CMD and CPPDD. Its cell membrane localisation resembled the CPPDD-associated mutant P5L but we measured a sustained decrease in ePPi similar to the CMD associated mutant S375del. Early upregulation of *ENPP1* under low ePPi concentrations further supports this to be a loss-of-function mutation. Hence, since the mutant successfully localised to the cell membrane, the glycine to arginine change at residue 389 appears to cause a loss of transport channel function, which caused the decrease in ePPi and increase in mineralization, although small, reflecting its milder phenotype. The involvement of G389R in autophagy also suggests that it undergoes mis-folding or some other abnormal processing, but perhaps to a lesser degree than E490del.

### E490del

The deletion of a glutamate residue at the C-terminus renders it unable to insert effectively into the cell membrane. However, this mutant functioned similar to wt. ANKH in regulating ePPi by increasing *ENPP1*, *TNAP* and *PIT-1* but was still pathogenic as it increased mineralization. We suggest that this mutant is recognised as a non-functional protein and is constantly recycled by the autophagy machinery, which consequently due to its loss also upregulates *ENPP1*, *TNAP* and *PIT-1* as compensation for the loss of ANKH at the cell membrane. It is noteworthy that the clinical presentation of CPPDD associated with the E490del is less severe than other forms that might be due to some of the ANKH getting to the membrane but at lower efficiency.

In summary, the corresponding pathogenetic mechanisms for each of the ANKH terminal CPPDD and central CMD associated mutations appear to be unique even between mutants causing similar phenotypes which are potentially associated with the levels of disease severity. They include effects on intracellular trafficking of ANKH, PPi transport channel function, mineralization, ENPP1 activity regulation, autophagy and guiding effects in expression level of *ENPP1, TNAP* and *PIT-1*, some of which could represent tractable targets for therapy.

## Methods

### Cell culture and plasmid transfection

CH-8 articular chondrocyte and HEK293 cells were cultured in high glucose DMEM supplemented with 10% FBS and 1% penicillin-streptomycin. Transfections were performed using FugeneHD (Promega, Wisconsin, USA). Transfections were optimised by counting the number of GFP expressing cells in at least 3 distinct fields of views and expressed as a percentage of transfected vs. non-transfected cells (Fig. S4).

### ANKH construct and mutant generation

Standard restriction cloning was used to create NM_054027.4:*ANKH-eGFP_N3* (*wt.ANKH)*) and NM_054027.4:*ANKH-eGFP_C1* (wt.ANKH_C1) overexpression constructs. A Q5 Site-Directed mutagenesis kit (New England BioLabs, Massachusetts, USA) was used to create the desired overexpression mutants with the following primers; P5L: 5′-GTGAAATTCCTGGCGCTCACG, 3′–CATGGAATTCGAAGCTTGAGC; E490del: 5′–AATGAAAATGGATCCATCGC, 3′–CTCTCTCATTTCCACGATG; S375del: 5′–TTCTTCCCAGTTCCAGTC, 3′–GAAGATCCGCAAAGGAAC; G389R: 5′–GCATCTCACCAGGTGGCTGATG, 3′–GCCCTCACTGTGACTGGA. For all experiments wt.ANKH was measured against empty eGFP construct and mutants against wt.ANKH.

### Confocal Imaging

CH-8 and HEK293 cells were cultured on 35 mm high glass bottom u-dishes (iBidi, Martinsried, Germany). Before visualisation, cells were PBS washed and fresh growth media added. Nuclear DNA was stained with Hoechst 33342 (5 ug/ml) (Thermo Fisher) for 10 mins. Confocal imaging was performed on a Zeiss LSM 880 with Airyscan with a temperature and CO_2_ controlled chamber. Images were processed on proprietary Zen 2.1 (Zeiss, Jena, Germany).

### Autophagy induction and rescue

HEK293 cells were grown to 60% confluence and co-transfected with *pmRFP_LC3* (autophagy marker, red) and wt. or mutant *ANKH_eGFP*. Autophagy was induced for three hours, replacing with fresh culture media, supplemented with 250 nM rapamycin, without essential amino acids. Cells were rescued at 24 hours in normal growth media and imaged. Intensity over distance was measured for co-localisation of mutants with LC3 in Zen through overlapping puncta, measuring raw arbitrary intensity plotted against distance^28^. Samples were blinded and cell counting was performed via tiling an 8 × 8 (1.68 mm^2^) area and counting transfected expressed cells (n > 100) recording cells with either cell membrane expression or cytoplasmic expression only, reported as a percentage of cells with only cell membrane expression.

### Inorganic pyrophosphate assay

Culture media from transfected and control CH-8 and HEK293 cells were collected every 24 hours and the concentration of PPi measured using the PPiLight Pyrophosphate Detection Kit (Lonza, Basel, Switzerland). Arbitrary luminescence was recorded on a MicroBeta TriLux 1450 (Perkin Elmer, Massachusetts, USA) reader using a 0.1 s integrated read time^29,30^. Results were normalised to the native non-cellular fluctuation in media only and concentration was deduced by plotting a standard curve.

### Alizarin red S mineralization assay

CH-8 cells were cultured to 60% confluence and transfected with wt.ANKH, mutants or GFP only plasmid, The media was replaced with mineralization media consisting of high glucose DMEM supplemented with 10% FBS, 50 ug/ml Ascorbic acid, 10 mM B-glycerol phosphate (BGP) and 100 nM dexamethasone 24 hours after transfection and the cells were incubated for another 48 hours. The mineralization measured by ARed-Q (Sciencecell Research Laboratories, CA, USA).

### Quantitative PCR

Transfected CH-8 and HEK293 cells were scraped at 24, 48 and 72 hours and RNA extracted by RNeasy Kit (Qiagen, Hilden, Germany). cDNA was synthesized using iScript and qPCR performed using SYBR Green Kits (both Bio-Rad, California, USA). QPCR Primers *ENPP1*: F–GGTGGACTTCTTCCTGTTATTA, R–GGTGACAATGCTGTAGTGA; *TNAP*: F–GCTTGACCTCCTCGGAAGAC, R–GGGGCCAGACCAAAGATAGA; *PIT-1*: F–GACTGGCGTCTCTTTCGTA, R–ATGATGGCAGCACTGATAAC; *ACTB*: F–TGACCCAGATCATGTTTGAGA, R-TACGGCCAGAGGCGTACAGC.

### ENPP1 enzyme activity assay

Transfected CH-8 cells were harvested as pellets via scraping at 24, 48 and 72 hours. The colourimetric substrate *p-*nitrophenyl thymidine 5′-monophosphate (Sigma, Welwyn Garden City, UK) was assessed following a published protocol^24^.

### Co-immunoprecipitation (co-IP) and western blotting

CH-8 and HEK293 cells were co-transfected with relevant plasmid at 60% confluence. Co-IP was performed using GFP-Trap magnetic agarose beads that implement a unique camelid nanobody (Chromotek, Martinsried, Germany). SDS PAGE was performed on the lysates and pull downs in 4–20% TGX protein gels, transferred to a PVDF membrane, blocked for 1 hour in 5% milk/TBS/0.1% tween20 and probed with 1:2500 anti-GFP antibody (NB600-308, R&D Systems, Abingdon, UK), 1 µg/ml anti-LC3 (NB100-2220, R&D Systems) and 1:5000 anti-GAPDH (AB128915, Abcam) overnight at 4 °C in 5% milk/TBST. Goat anti-rabbit IgG-HRP (AB97051, Abcam, Cambridge, UK) 1:5000 secondary was incubated for 1 hour in 5% milk/TBST at room temperature and detected using Clarity ECL western substrate on a ChemiDoc XRS + (all Bio-Rad). Full gels are in the Supplementary Figs. 5–7.

### Statistical analysis

All experiments were repeated a minimum of three times. For the chemical data analysis, wt.ANKH effect was compared to null vector control whilst mutant effect was compared to wt.ANKH. Assuming equal variance, 2-tailed t-test was used to evaluate significance for normally distributed data, whilst a Mann-Whitney *u*-test was performed on non-normally distributed data. An *F*-test was performed to check equal and unequal variance. Statistical significance was set at *p* < 0.05. We use # to highlight the data 0.05 < p < 0.1, *p < 0.05, **p < 0.01, ***p < 0.0005.

## Supplementary information

Supplementary information.

Supplementary movie S1.

Supplementary movie S2.

Supplementary movie S3.

Supplementary movie S4.

## Data Availability

Materials and data are available with reasonable request from the corresponding author.
