# Supplementary material for: Differences in intracellular localisation of ANKH mutants that relate to mechanisms of calcium pyrophosphate deposition disease and craniometaphyseal dysplasia
Source: Sci Rep. 2020 May 4;10:7408. doi: 10.1038/s41598-020-63911-x (PMC7198517; doi:10.1038/s41598-020-63911-x)
Supplement: Supplementary file 1 — Supplementary information. [file 41598_2020_63911_MOESM1_ESM.pdf]

Intracellular deviations of ANKH mutants indicating mechanisms of calcium pyrophosphate deposition disease and craniometaphyseal dysplasia

Sunny Vijen[1], Chris Hawes[1 †], John Runions[1], R. Graham Russell[2], B. Paul Wordsworth[2], Andrew J. Carr[2], Ryan C. Pink[1*], Yun Zhang[1*]

1. Department of Biology and Medical Sciences, Oxford Brookes University, Gipsy Lane, Oxford OX3 0BP

2. Nuffield Department of Orthopaedics, Rheumatology and Musculoskeletal Sciences, University of Oxford Institute of Musculoskeletal Sciences, Windmill Road, Oxford OX3 7HE

**Corresponding authors:*

- Ryan C. Pink, Department of Biology and Medical Sciences, Oxford Brookes University, Gipsy Lane, Oxford OX3 0BP. rpink@brookes.ac.uk. +441865483607
- Yun Zhang, Department of Biology and Medical Sciences, Oxford Brookes University, Gipsy Lane, Oxford OX3 0BP. yun.zhang@brookes.ac.uk. +447796365618

[†] *Deceased 4 July 2019.*

Supplementary Information

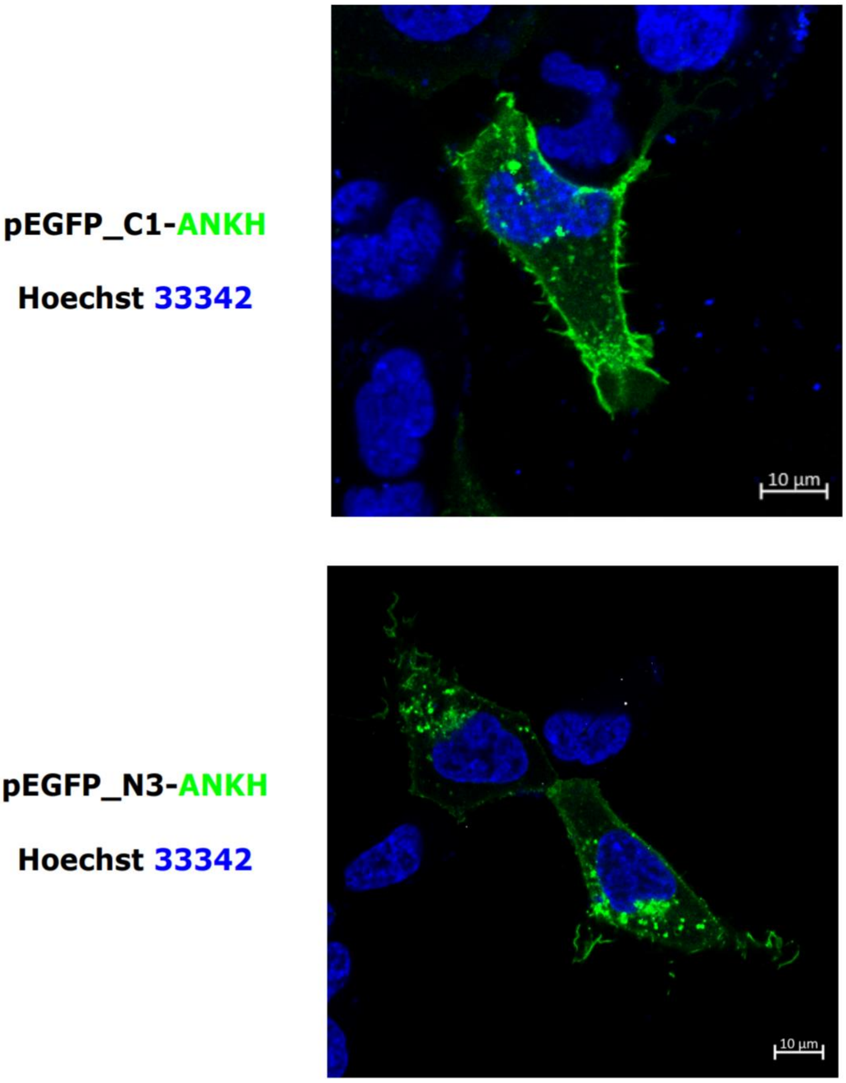

Figure S1. *Wt. ANKH N3 and Wt. ANKH C1 are both specifically expressed at cytoplasmic membrane.*

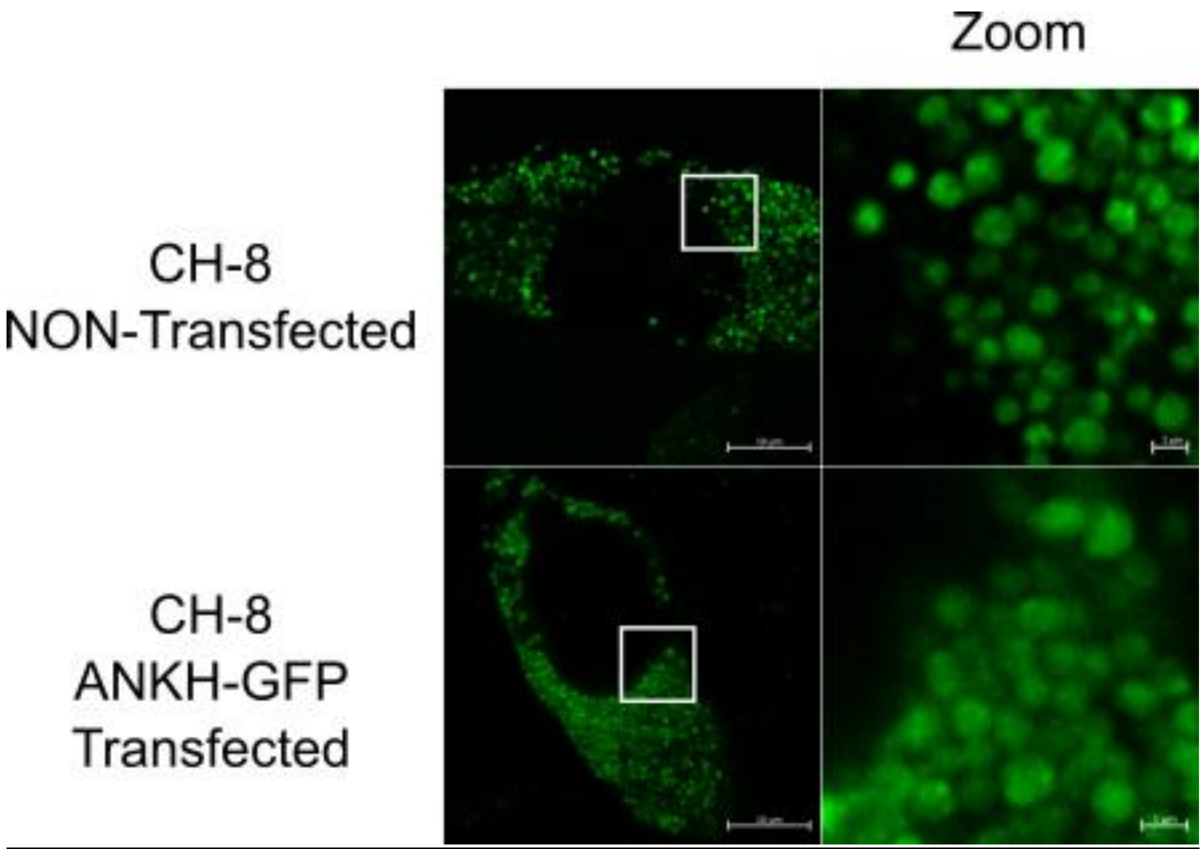

Figure S2. *CH-8 cells displayed auto-fluorescence and showed large punctate like structures that masked overexpression of control eGFP and wt.ANKH-eGFP.*

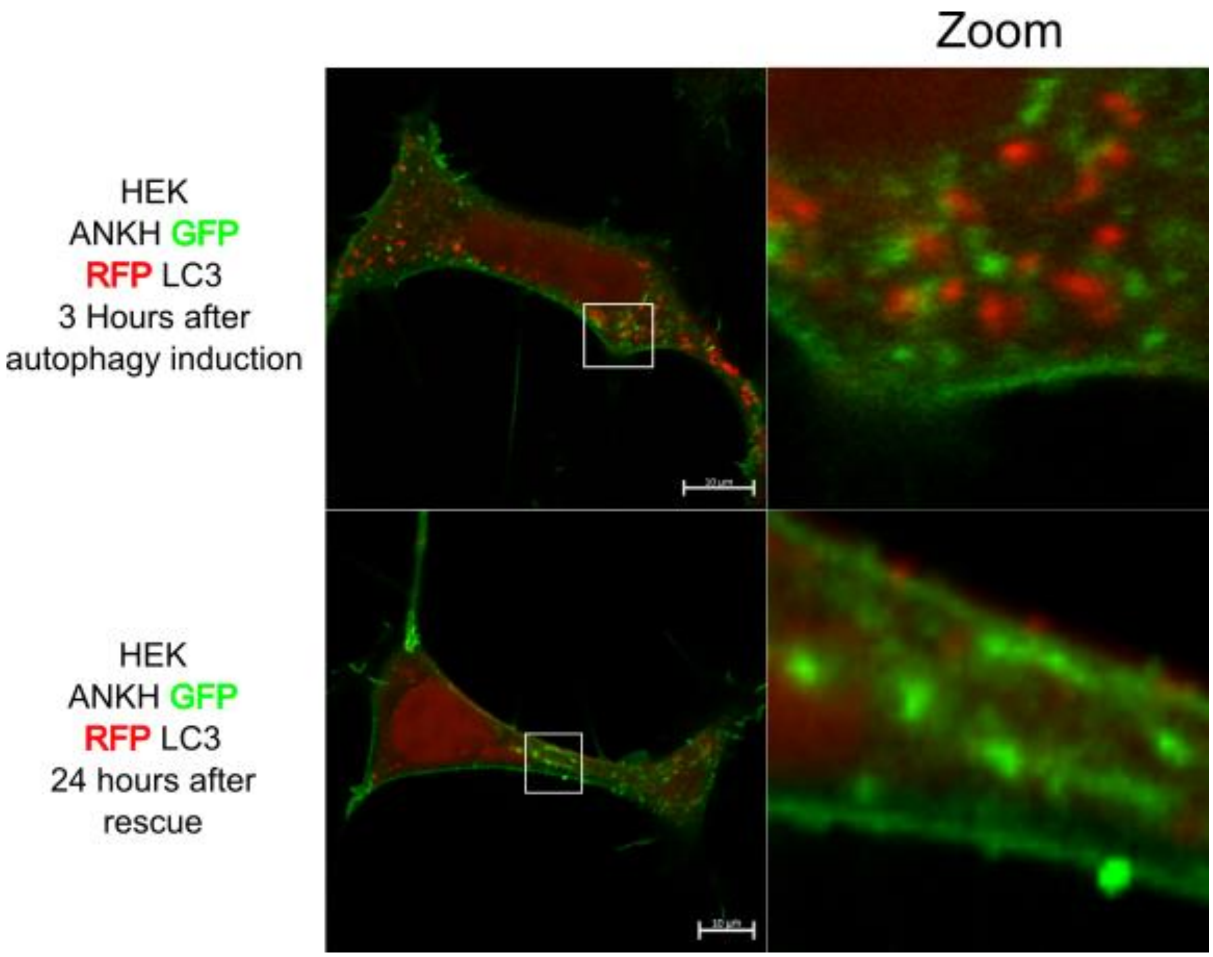

Figure S3. *Wt.ANKH-eGFP did not co-localise with pmRFP-LC3 under autophagic or rescue conditions in HEK293 cells.*

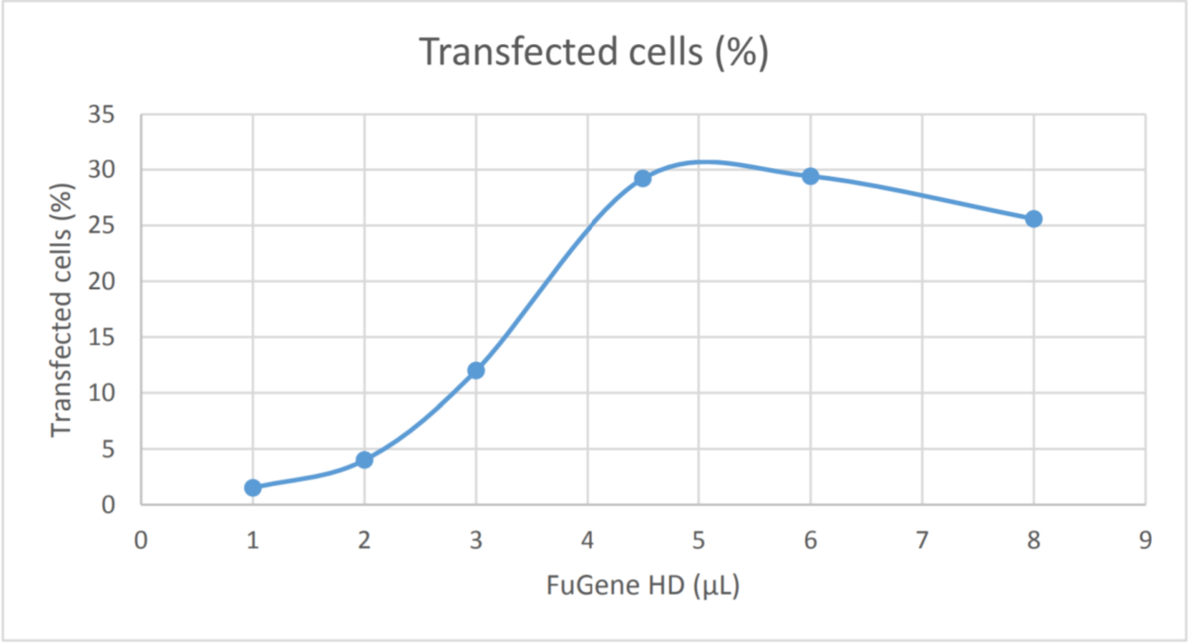

Figure S4. *Optimisation of wt.ANKH-GFP transfection optimisation in HEK293 cells.*

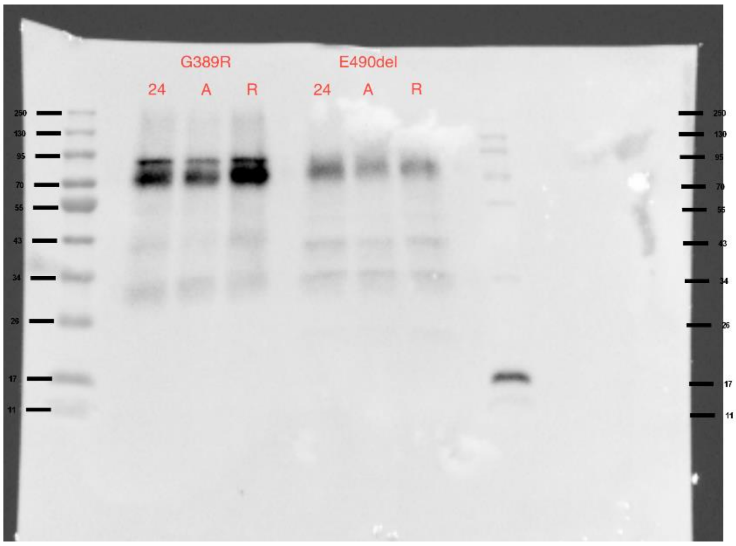

Figure S5. *HEK293 Lysates of G389R and E490del ANKH-GFP (rep of three) at 24H, with autophagic media for 3 h (A), with normal growth media for 24H (R) anti-GFP full gel Western blot of IP GFP pull down cropped in figure 4I and 5I.*

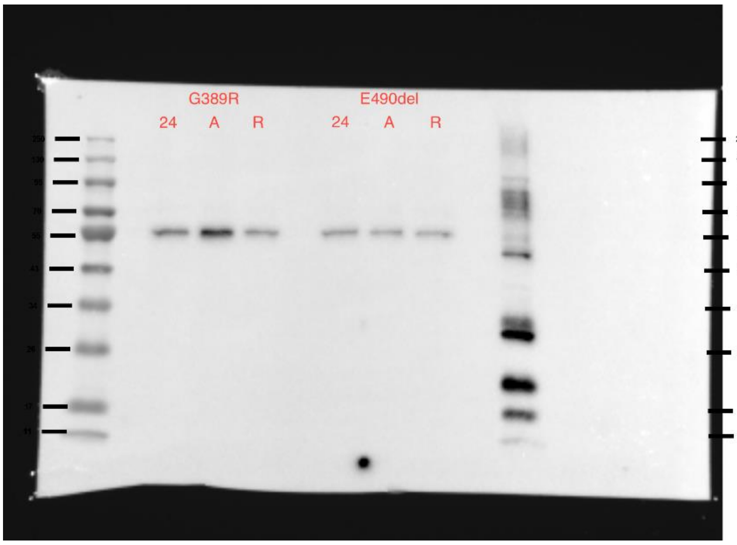

Figure S6. *HEK293 Lysates of G389R and E490del ANKH-GFP (rep of three) at 24H, with autophagic media for 3 h (A), with normal growth media for 24H (R) anti-LC3 Western blot of IP GFP pull down cropped in figure 4I and 5I.*

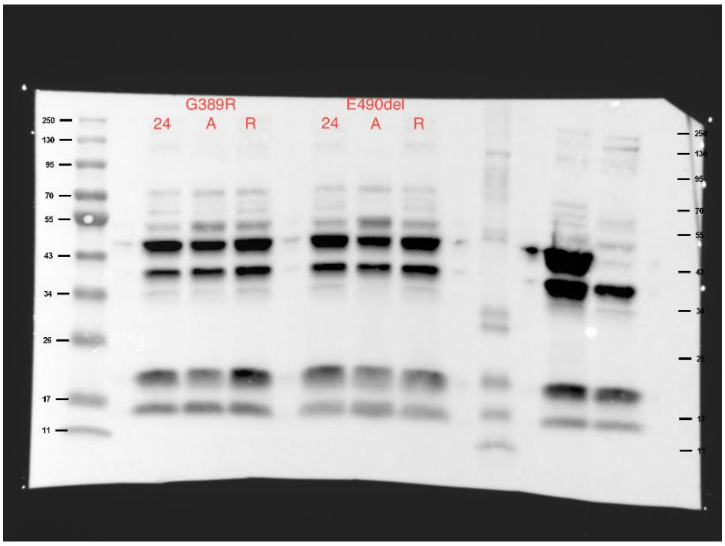

Figure S7. *HEK293 Lysates of G389R and E490del ANKH-GFP (rep of three) at 24H, with autophagic media for 3 h (A), with normal growth media for 24H (R) anti-GFP full gel Western blot of whole cell cropped in figure 4I and 5I.*

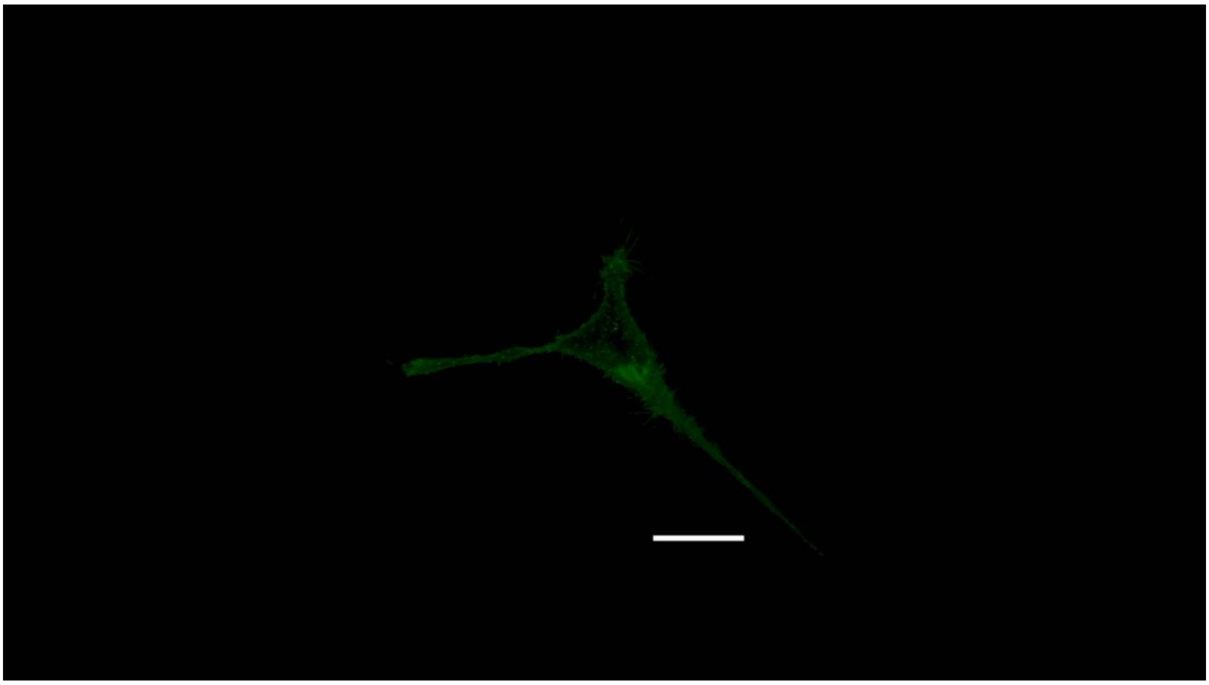

Movie S1*. ANKH localisation to the cell membrane was non-uniform.*
Short link title: ANKH localisation was nonuniform

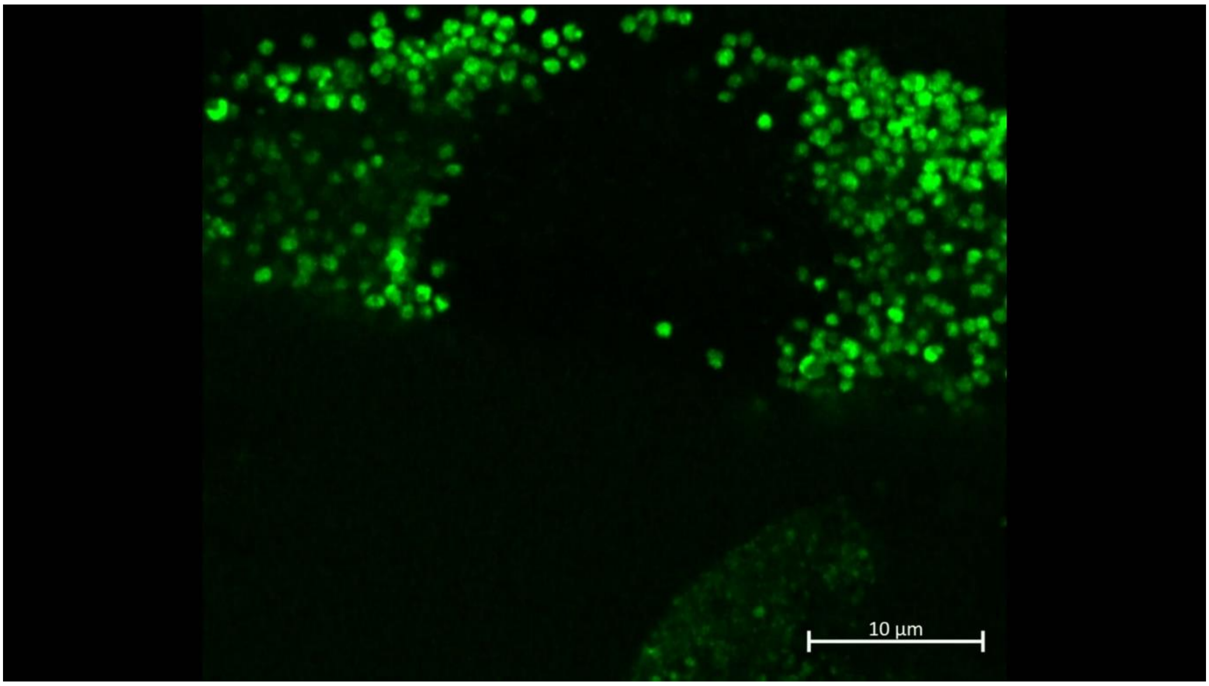

Movie S2. *CH-8 cells displayed auto-fluorescence and showed large punctate like structures that masked overexpression of control eGFP and wt.ANKH-eGFP.*
Short link title: CH8 autofluorescence and punctates

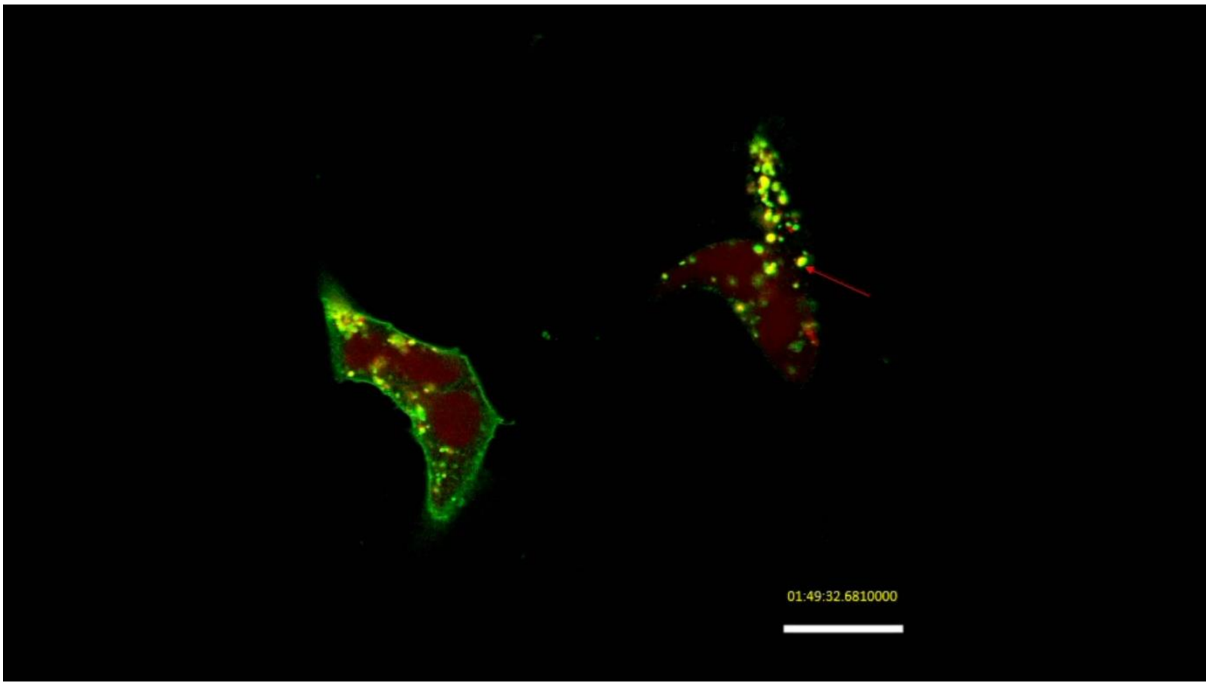

Movie S3. *Co-localisation of G389R-eGFP with RFP-LC3 under autophagy rescue condition in HEK293 cells.*
Short link title: Colocalisation of G389R with LC3

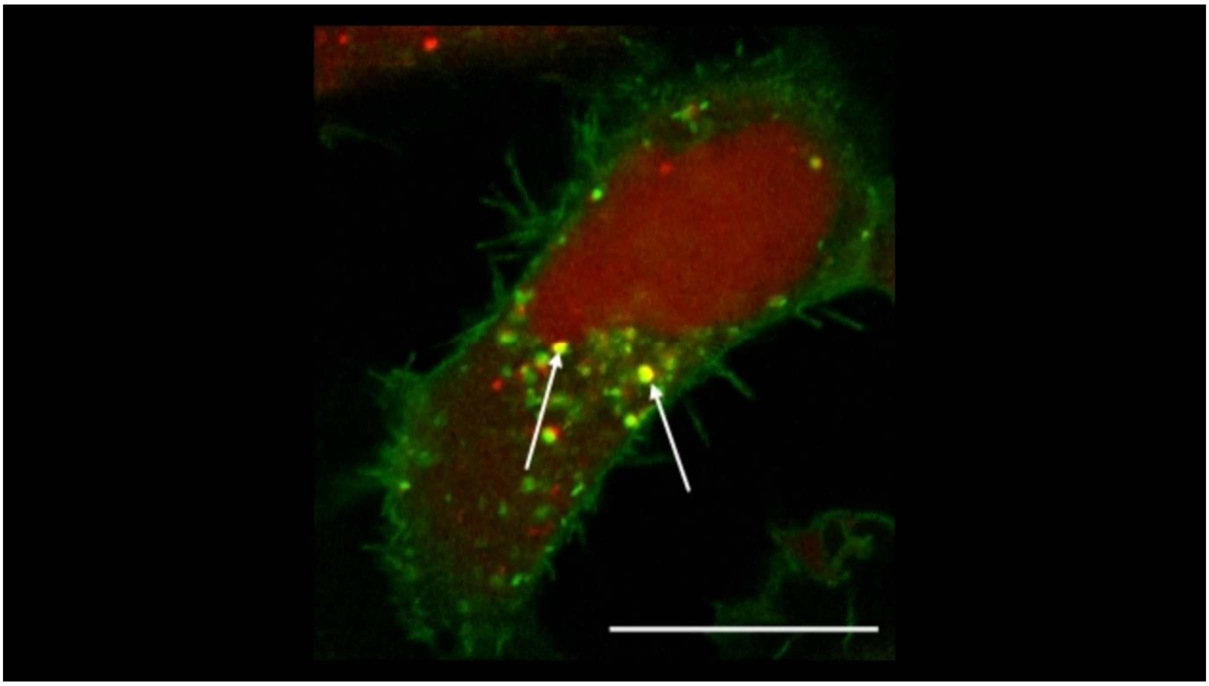

Movie S4. *Co-localisation of E490del-GFP with RFP-LC3 under autophagy rescue condition in HEK293 cells.*
Short link title: Colocalisation of E490del with LC3
